# Supplementary material for: Effect of Nano-Selenium on Nutritional Quality of Cowpea and Response of ABCC Transporter Family
Source: Molecules. 2023 Feb 1;28(3):1398. doi: 10.3390/molecules28031398 (PMC9921613; doi:10.3390/molecules28031398)
Supplement: Supplementary file 1 [file molecules-28-01398-s001.zip › molecules-2079584-supplementary.pdf]

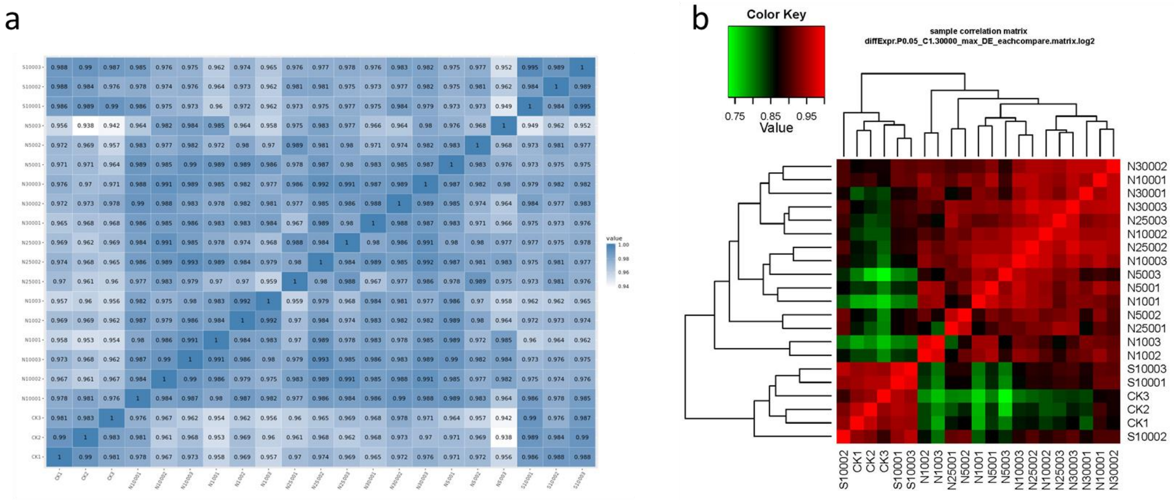

Figure S1. The heat map of correlation analysis of data among cowpea transcriptome samples.

a. Heat map of Pearson correlation coefficient between samples.

b. Sample correlation matrix.

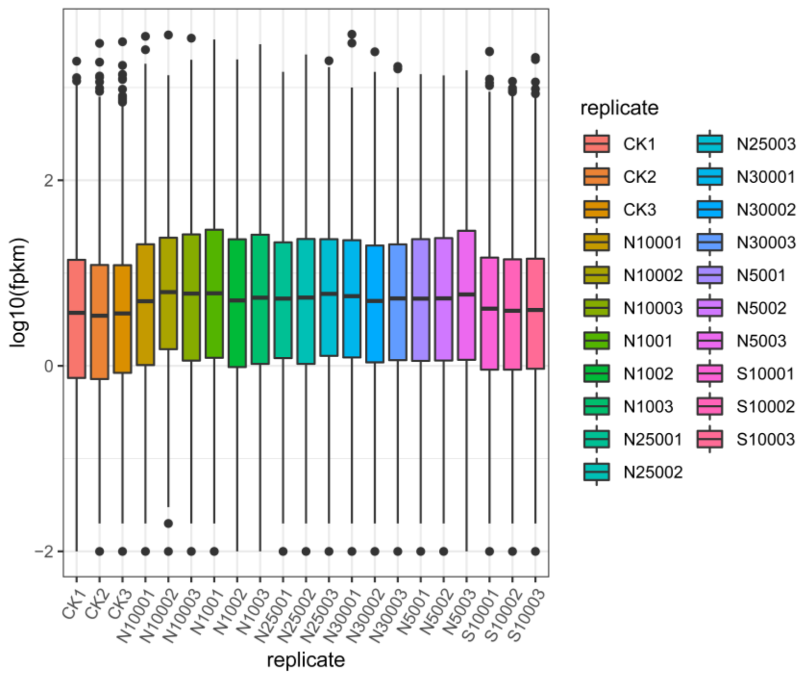

Figure S2. Density distribution of cowpea transcriptome data.

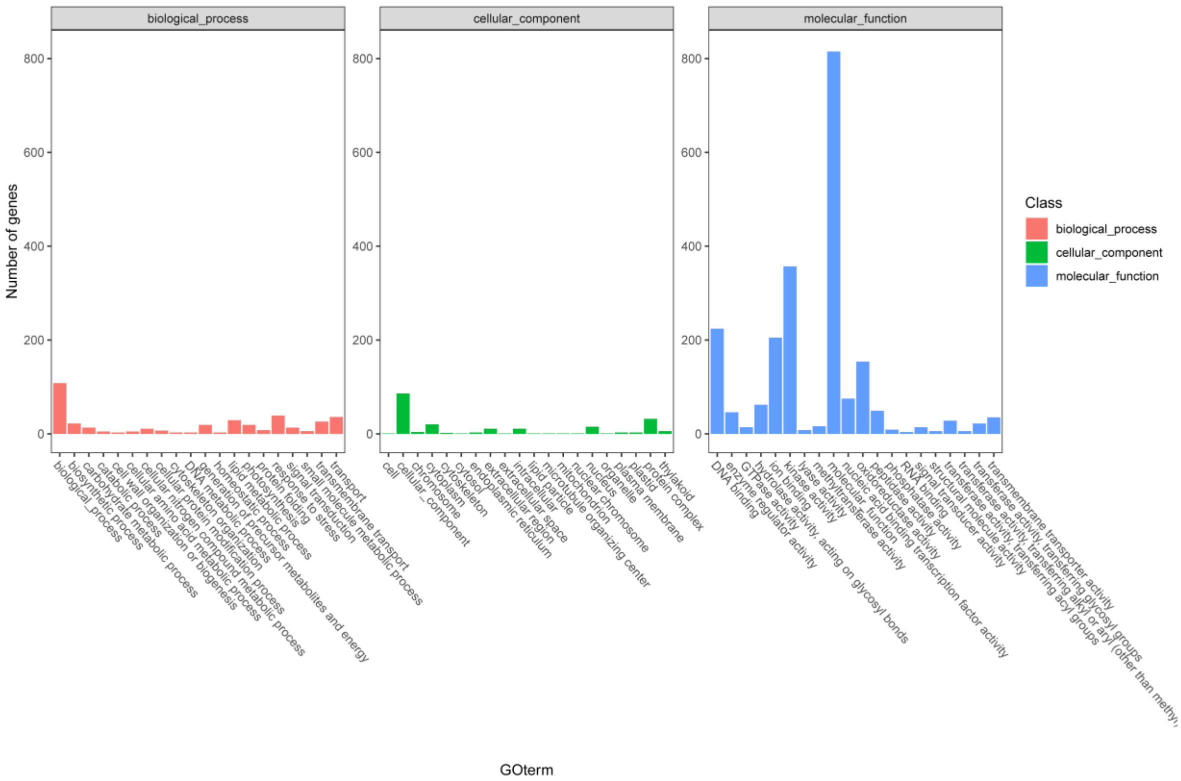

Figure S3. GO enrichment analysis of differentially expressed genes in cowpea samples.

Table S1. Analysis on the number and proportion of differentially expressed genes in ABC gene family of cowpea in different treatment groups.

Treatment sample	Total	Up/Down-regulated	Number and total proportion of differential expression of ABCB, ABCC, ABCG subfamilies			
			ABCB	ABCC	ABCG	Total proportion
CK-N100	102	51/51	25	13	41	77.5 %
CK-N500	98	49/49	24	13	38	76.3 %
CK-N1000	104	55/49	25	13	43	77.8 %
CK-N2500	102	56/46	26	13	40	77.5 %
CK-N3000	103	44/59	25	13	42	77.7 %
CK-S1000	102	60/42	25	13	40	76.5 %
N100-N500	100	52/48	24	13	40	77.0 %
N100-N1000	102	54/48	24	13	42	77.5 %
N100-N2500	102	54/48	24	13	42	77.5 %
N100-N3000	100	44/56	24	13	40	77.0 %
N500-N1000	102	54/48	24	13	42	77.5 %
N500-N2500	101	50/51	25	13	40	77.2 %
N500-N3000	101	49/52	24	13	41	77.2 %
N1000-N2500	103	50/53	25	13	42	77.7 %
N1000-N3000	104	45/59	25	13	43	77.9 %
N2500-N3000	102	41/61	25	13	41	77.5 %
N1000-S1000	103	54/49	24	13	43	77.7 %

Table S2. Treatment concentration and sampling tissue of cowpea.

Sample name	Reagent	Treatment concentration (mmol/L)	Purpose	Tissue
CK	H_2O	0	Physiological indicators and transcriptome	Young leaves and pods
N100	SeNPs	0.1	Physiological indicators and transcriptome	Young leaves and pods
N500	SeNPs	0.5	Physiological indicators and transcriptome	Young leaves and pods
N1000	SeNPs	1.0	Physiological indicators and transcriptome	Young leaves and pods
N2500	SeNPs	2.5	Physiological indicators and transcriptome	Young leaves and pods
N3000	SeNPs	3.0	Physiological indicators and transcriptome	Young leaves and pods
S100	Na_2SeO_3	0.1	Physiological indicators	Pods
S500	Na_2SeO_3	0.5	Physiological indicators	Pods

S1000	Na_2SeO_3	1.0	Physiological indicators and transcriptome	Young leaves and pods
S2500	Na_2SeO_3	2.5	Physiological indicators	Pods
S3000	Na_2SeO_3	3.0	Physiological indicators	Pods
